# Management of local recurrence after radical nephrectomy: surgical removal with or without systemic treatment is still the gold standard. Results from a multicenter international cohort

**DOI:** 10.1007/s11255-021-02966-9

**Published:** 2021-08-21

**Authors:** Michele Marchioni, Petros Sountoulides, Maria Furlan, Maria Carmen Mir, Lucia Aretano, Jose Rubio-Briones, Mario Alvarez-Maestro, Marta Di Nicola, Alfredo Aguilera Bazán, Alessandro Antonelli, Claudio Simeone, Luigi Schips

**Affiliations:** 1 Department of Medical, Oral and Biotechnological Sciences, G. D’Annunzio University of Chieti, Urology Unit, SS Annunziata Hospital, Chieti, Italy; 2 Department of Urology, General Hospital of Veria, Veria, Greece; 3 Urology Unit, ASST Spedali Civili of Brescia, Department of Medical and Surgical Specialties, Radiological Science and Public Health, University of Brescia, Brescia, Italy; 4 Department of Urology, Instituto Valenciano de Oncologia, Valencia, Spain; 5 Department of Urology, Hospital Universitario La Paz, Madrid, Spain; 6 Department of Medical, Oral and Biotechnological Sciences, G. d’Annunzio University of Chieti, Chieti, Italy; 7 Department of Urology, Azienda Ospedaliera Universitaria Integrata of Verona, University of Verona, Verona, Italy; 8 Department of Medical, Oral and Biotechnological Sciences, Laboratory of Biostatistics, University “G. d’Annunzio” Chieti-Pescara, Via dei Vestini, Campus universitario, 66100 Chieti, Italy

**Keywords:** Renal cell carcinoma, Retroperitoneal recurrence, Renal cancer treatment, Renal cancer surgery, Radical nephrectomy, Renal fossa recurrence

## Abstract

**Objective:**

To evaluate the survival outcomes of patients with local recurrence after radical nephrectomy (RN) and to test the effect of surgery, as monotherapy or in combination with systemic treatment, on cancer-specific mortality (CSM).

**Methods:**

Patients with local recurrence after RN were abstracted from an international dataset. The primary outcome was CSM. Cox’s proportional hazard models tested the main predictors of CSM. Kaplan–Meier method estimates the 3-year survival rates.

**Results:**

Overall, 96 patients were included. Of these, 44 (45.8%) were metastatic at the time of recurrence. The median time to recurrence after RN was 14.5 months. The 3-year cancer-specific survival rates after local recurrence were 92.3% (± 7.4%) for those who were treated with surgery and systemic therapy, 63.2% (± 13.2%) for those who only underwent surgery, 22.7% (± 0.9%) for those who only received systemic therapy and 20.5% (± 10.4%) for those who received no treatment (*p* < 0.001). Receiving only medical treatment (HR: 5.40, 95% CI 2.06–14.15, *p* = 0.001) or no treatment (HR: 5.63, 95% CI 2.21–14.92, *p* = 0.001) were both independently associated with higher CSM rates, even after multivariable adjustment. Following surgical treatment of local recurrence 8 (16.0%) patients reported complications, and 2/8 were graded as Clavien–Dindo ≥ 3.

**Conclusions:**

Surgical treatment of local recurrence after RN, when feasible, should be offered to patients. Moreover, its association with a systemic treatment seems to warrantee adjunctive advantages in terms of survival, even in the presence of metastases.

**Supplementary Information:**

The online version contains supplementary material available at 10.1007/s11255-021-02966-9.

## Introduction

Kidney cancer is the sixth most common cancer among men and the eighth among women, usually diagnosed as localized disease and with a generally good prognosis[1]. Depending on several patients, treatment, and tumor’s characteristics local recurrence (LR) rates after radical nephrectomy (RN) widely range [2–4], with some series reporting LR rates up to 28.0% [2].

After RN, tumor might recur in the soft tissue of the ipsilateral renal fossa, adrenal gland, retroperitoneal lymph node tissue, and psoas muscle, with or without evidence of other metastatic sites [5–10]. European Association of Urology (EAU) guidelines recommend to perform surgery in all patients with no adverse prognostic factors [2]. However, this recommendation is mostly supported by evidence from few retrospective studies [3]. In addition, the use of systemic treatments in patients with LR is also debated, in particular in those with high-risk features and high disease burden [2].

With this in mind, we aimed to evaluate the survival outcomes of patients with LR after RN and to test the effect of local recurrence surgery (LRS), as monotherapy or in combination with systemic treatment, on cancer-specific mortality (CSM).

## Materials and methods

### Study population and main characteristics of interest

Data on patients with LR following RN for renal cell carcinoma (RCC) were retrieved from a purpose-built international database including tertiary academic centers from Italy and Spain. Primary RN was performed in all patients affected by non-metastatic RCC with a curative intent. LR was defined as any recurrence in the renal fossa, ipsilateral adrenal gland, retroperitoneal lymph nodes or inferior vena cava. As in previous studies on this topic, oligometastatic patients (with three or fewer metastases) at the time of LR were also included [2]. The dataset included data concerning the primary tumor and RN, as well as information about LR and its treatment (LRS, systemic therapy, combination of LRS and systemic therapy, expectant management).

The main clinical characteristics of interest at the time of RN were patients’ age and gender, RN year, Charlson comorbidity index, RN approach (laparoscopic or open), ipsilateral adrenalectomy or lymph node dissection and positive surgical margin status. The main pathologic characteristics from RN specimens were pathological T-stage, pathological N-stage, histological subtype [clear cell renal cell carcinoma (ccRCC) or non-ccRCC], tumor grade, presence of sarcomatoid differentiation, and necrosis.

The main clinical and surgical characteristics of interest at the time of LR were size, number and site of recurrences, the time interval from RN to the diagnosis of recurrence (< 24 vs. ≥ 24 months), symptomatic or asymptomatic LR and LR treatment. The presence of metastases was noted at the time of recurrence and during the follow-up. Among those who underwent LRS the following features were noted: surgical approach (open vs. laparoscopic), complications rate according to the Clavien–Dindo classification, positive surgical margin status after surgery, LR histology (ccRCC or non-ccRCC). Moreover, RCC recurrences tumor grade was assigned to LR and when available was reported.

After RN all patients underwent chest and abdomen CT-scan or abdomen ultrasound according to individual risk stratification, as per the EAU guidelines [11]. All LR were confirmed with a CT-scan or MRI. After LR, all patients were followed with chest and abdomen imaging every 3 months for the first 2 years and twice a year thereafter.

### Primary and secondary outcomes

The study’s primary outcome was CMS following LR. We defined the follow-up as the time interval from LR to death due to cancer. Patients who did not die from cancer were censored at the time of death for other causes or to the last follow-up visit available. Secondary outcomes of interest were metastatic status at LR, time to recurrence after RN and progression-free survival. Progression was defined as the development of metastases in those who were classified as M0 at the time of LR. Moreover, progression was also defined by death due to the cancer for either patients with or without metastases at the time of LR. Therefore, progression-free survival was defined as the time interval from LR to progression ascertainment.

### Statistical analyses

Descriptive statistics relied on frequencies and percentages for categorical variables and on median and interquartile ranges (IQR) for continuous variables. The analyses included several steps. First, we stratified our cohort according to the metastatic status at the time of LR (M + vs. M0). Similarly, we stratified our cohort according to the LRS status (patients in whom surgery was performed vs. those in whom was not). Kruskal Wallis rank sum test and Chi-square test were used to ascertain differences in medians and proportions, respectively.

Second, univariable and multivariable logistic regression models tested main predictors of metastatic status at LR. Third, median recurrence-free survival after RN as well as progression-free survival and cancer-specific survival after LR were estimated with the Kaplan–Meier method. Fourth, univariable and multivariable Cox’s proportional hazard regression models tested main predictors of CSM. Finally, we tested the interaction effect between LR treatment and M + status at recurrence. In all multivariable models, we included covariates that were statistically significant at univariable analyses.

All statistical analyses were performed using R Statistical Software (version 4.0.0; R Foundation for Statistical Computing, Vienna, Austria). All tests were two-tailed, and a *p* value < 0.05 was considered indicative of a statistically significant association.

## Results

### Main characteristics of included patients

Overall, 96 patients with LR after RN were included. Recurrences were more frequently localized in the renal fossa (50.0%) and in 44 patients (45.8%) LR was associated with distant metastases (M + stage). The median age at RN was 63.0 (IQR 52.3–72.0) years and most of RN were performed before 2004 (53.1%). Most of the patients were males (65.6%) with a Charlson Comorbidity index 0–1 (82.5%) and no symptoms at the time of LR (92.7%). The majority of RNs were open (79.2%) without adrenalectomy (56.4%) and without lymph node dissection (56.4%). Primary tumors were more frequently pT3 (63.5%), pNx (61.5%), ccRCC (71.9%) of grade 3–4 (76.9%). Sarcomatoid differentiation was reported in 8.6% of cases. Only 7.3% of patients had positive surgical margins after RN. The median number of recurrences was 2 (IQR 1.0–2.0) with a median size of 4.0 (IQR 3.0–6.0) cm. Of all, 50 patients (52.1%) received LRS while 16 of the surgically treated patients (32.0%) received systemic therapy as well.

### Main characteristics of metastatic patients

Patients who had a RN before 2004 were more frequently diagnosed as M + at the time of LR (65.9 vs. 42.3%, *p* = 0.021; Supplementary Material 1). Moreover, patients with renal fossa LR were also more frequently M + (63.6 vs. 38.5%, *p* = 0.014). Patients with metastatic disease were more likely to not receive surgical treatment (79.5 vs. 21.2%, *p* < 0.001) but only systemic therapy (43.6 vs. 15.4%) or expectant management (35.9 vs. 10.3%; *p* < 0.001; Supplementary Material 2). Within logistic regression models, after multivariable adjustment, age between 55–70 years (OR: 3.02, 95% CI: 1.05–9.30, *p* = 0.045) and laparoscopic RN (OR: 0.21, 95% CI: 0.04–0.77, *p* = 0.030) were statistically significant predictors of M + status (Supplementary Material 3).

### Main characteristics of surgically treated patients and surgical outcomes

Patients who underwent LRS were younger (median age 61.0 vs. 66.5 years, *p* = 0.028) and more likely to had LR within the first 24 months from RN (52.0 vs. 71.7%, *p* = 0.047). Moreover, surgically treated patients had more often laparoscopic RN (36.0 vs. 4.3%, *p* < 0.001), but less frequently RN and adrenalectomy (32.7 vs. 55.6%, *p* = 0.025) or lymph node dissection (20.4 vs. 41.3%, *p* = 0.027). Recurrences were more frequently treated when localized at the ipsilateral adrenal gland (20.0 vs. 2.2%, *p* = 0.006) and less frequently when localized in the renal fossa (34.0 vs. 67.4%, *p* = 0.001) or other sites (2.0 vs. 13.0%, *p* = 0.038; Table 1). Complications occurred in 8 patients (16.0%) and 2 were classified as Clavien–Dindo grade III (25.0%). Histology showed a non-ccRCC at the site of recurrence in 24.0% of patients and 58.0% of all LR were of grade 3–4 (Table 2).

**Table 1:** Main patients’ characteristics at radical nephrectomy and recurrence stratified according to the surgical treatment of recurrence

	No surgery of recurrence (*n* = 46)	Surgical treatment of recurrence (*n* = 50)	*p* value
Patients characteristics at radical nephrectomy			
Age (years)	66.5 (57.8, 73.0)	61.0 (49.2, 69.9)	**0.028**^**1**^
Age according to tertile (years)			**0.014**^**2**^
< 55	7 (15.2%)	21 (42.0%)	
55–70	25 (54.3%)	17 (34.0%)	
> 70	14 (30.4%)	12 (24.0%)	
Year of radical nephrectomy			0.062^2^
1988–2004	29 (63.0%)	22 (44.0%)	
2005–2018	17 (37.0%)	28 (56.0%)	
Time to recurrence ≥ 24 months			**0.047**^**2**^
< 24	33 (71.7%)	26 (52.0%)	
≥ 24	13 (28.3%)	24 (48.0%)	
Gender male	30 (65.2%)	33 (66.0%)	0.936^2^
Charlson comorbidity index > 1 (missing = 16)	7 (15.2%)	7 (20.6%)	0.532^2^
Symptomatic at recurrence	5 (10.9%)	2 (4.0%)	0.196^2^
Laparoscopic radical nephrectomy approach	2 (4.3%)	18 (36.0%)	** < 0.001**^**2**^
pT stage at radical nephrectomy			0.064^2^
T1–2	7 (15.2%)	18 (36.0%)	
T3	34 (73.9%)	27 (54.0%)	
T4	5 (10.9%)	5 (10.0%)	
pN stage at radical nephrectomy			0.284^2^
pN0	10 (21.7%)	12 (24.0%)	
pN1	10 (21.7%)	5 (10.0%)	
pNx	26 (56.5%)	33 (66.0%)	
Non-ccRCC at RN specimen	15 (32.6%)	12 (24.0%)	0.349^2^
Tumor grade 3–4 (missing = 5)	32 (74.4%)	38 (79.2%)	0.591^2^
Sarcomatoid dedifferentiation at RN	3 (6.5%)	4 (11.4%)	0.436^2^
Necrosis at RN (missing = 4)	5 (10.9%)	9 (19.6%)	0.246^2^
Radical nephrectomy associated with adrenalectomy	25 (55.6%)	16 (32.7%)	**0.025**^**2**^
Lymph node dissection at RN	19 (41.3%)	10 (20.4%)	**0.027**^**2**^
Positive surgical margins at RN	3 (6.5%)	4 (8.0%)	0.781^2^
Patients characteristics at recurrence			
Recurrence size (cm) (missing = 21)	4.9 (3.0, 6.2)	4.0 (2.8, 5.8)	0.576^1^
Number of recurrences (missing = 2)	1.0 (1.0, 2.0)	1.0 (1.0, 2.0)	0.074^1^
Recurrence in the renal fossa	31 (67.4%)	17 (34.0%)	**0.001**^**2**^
Recurrence at the psoas muscle	4 (8.7%)	8 (16.0%)	0.280^2^
Recurrence at ipsilateral adrenal gland	1 (2.2%)	10 (20.0%)	**0.006**^**2**^
Recurrence at lymph nodes	15 (32.6%)	22 (44.0%)	0.252^2^
Recurrence at other sites	6 (13.0%)	1 (2.0%)	**0.038**^**2**^
Systemic therapy after recurrence (missing = 18)	23 (56.1%)	16 (43.2%)	0.257^2^

Bold are reported statistically significant values

^1^Kruskal–Wallis rank sum test

^2^Pearson’s Chi-squared test

**Table 2:** Main surgical and pathological outcomes after local recurrence surgery

Main surgical and pathological outcomes after local recurrence surgery (*n* = 50)	
Associated systemic treatment (missing = 13)	16 (43.2%)
Laparoscopic surgery of recurrence	6 (12.0%)
Intra- and post-operative complications	8 (16.0%)
Highest complication grade according to Clavien–Dindo	
1	4 (50.0%)
2	2 (25.0%)
3	2 (25.0%)
Positive surgical margin at recurrence (missing = 14)	1 (2.8%)
Non-clear cell renal cell carcinoma	12 (24.0%)
Tumor grade 3–4 at recurrence (missing = 26)	14 (58.%)

### Survival outcomes

**Fig. 1:**
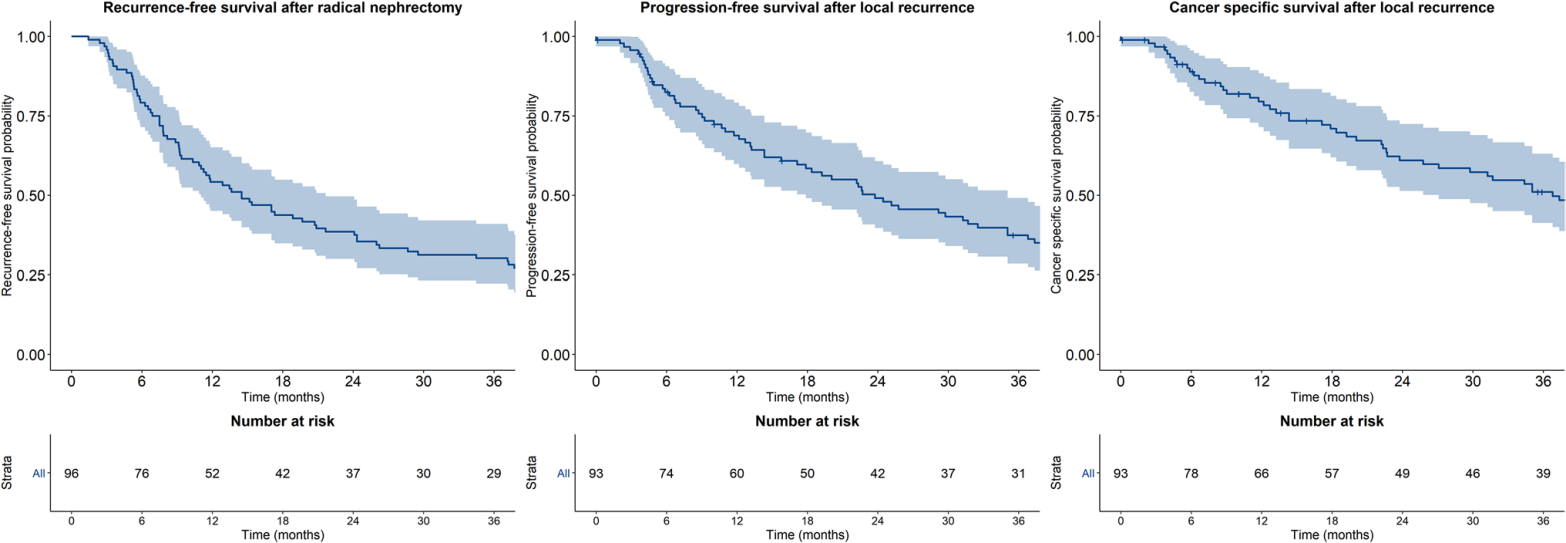
Kaplan–Meier curves showing recurrence-free survival rates after radical nephrectomy, progression-free survival rates after local recurrence and cancer-specific survival rates after local recurrence. Shadowed curve represents 95% confidence intervals

The median time from RN to LR was 14.5 (95% CI 11.0–21.7) months (Fig. 1) and the median follow-up of patients after LR was 30.4 (IQR: 10.1–69.3) months. While on follow up, 72 patients progressed (75.0%) and 72 (75.0%) died. Among those who experienced a progression 64 (88.9%) died. 81.9% of all deaths were cancer-related.

After LR the median time to progression was 23.8 (95% CI: 17.9–35.1) months and the median cancer-specific free survival was 36.8 (95% CI: 27.1–69.8) months (Fig. 1). The 3-year cancer-specific survival rates were 92.3% (± 7.4%) for patients treated with surgery and systemic therapy, 63.2% (± 13.2%) for those who only underwent surgery, 22.7% (± 0.9%) for those who were treated with systemic therapy only and 20.5% (± 10.4%) for those who received expectant management (*p* < 0.001). Interestingly, the 3-year cancer-specific survival rates for M0 patients at the time of LR were 71.8% (± 6.9%) with a median survival of 79.2 months (95% CI: 53.5-not reached). Receiving only medical treatment (HR: 5.40, 95% CI 2.06–14.15, *p* = 0.001) or expectant management (HR: 5.63, 95% CI 2.21–14.92, *p* = 0.001) were both independently associated with higher CSM rates, even after multivariable adjustment (Table 3). Furthermore, the interaction test between LRS and metastatic status at recurrence failed to show any statistically significant interaction (*p* = 0.393).

**Table 3:** Univariable and multivariable Cox proportional hazard models predicting cancer-specific mortality

	Univariable Cox regression Hazard ratio (95% CI, *p* value)	Multivariable Cox regression Hazard ratio (95% CI, *p* value)
Age at radical nephrectomy		
< 55 years	Reference	–
55–70 years	1.82 (0.97–3.42, *p* = 0.063)	–
> 70 years	1.47 (0.73–2.97, *p* = 0.279)	–
Year of radical nephrectomy (2005–2018 vs. 1988–2004)	0.57 (0.33–0.97, *p* = 0.038)	0.67 (0.38–1.18, *p* = 0.164)
Time to recurrence (months)	1.00 (0.99–1.00, *p* = 0.340)	–
Gender (female vs. male)	0.92 (0.54–1.58, *p* = 0.765)	–
Recurrence size (cm)	1.01 (0.93–1.10, *p* = 0.819)	–
Number of recurrences	1.05 (0.78–1.41, *p* = 0.744)	–
Recurrence at renal fossa (yes vs. no)	2.26 (1.33–3.85, *p* = 0.003)	1.58 (0.90–2.79, *p* = 0.111)
Type of treatment after recurrence		
Combined	Reference	Reference
Only surgery	1.93 (0.66–5.59, *p* = 0.227)	1.84 (0.62–5.48, *p* = 0.272)
Only medical	6.67 (2.58–17.20, *p* < 0.001)	5.40 (2.06–14.15, *p* = 0.001)
Expectant management	6.10 (2.32–15.98, *p* < 0.001)	5.63 (2.12–14.92, *p* = 0.001)
Unknown	1.41 (0.50–3.99, *p* = 0.516)	1.88 (0.64–5.51, *p* = 0.248)
Metastatic status (M + vs. M0)	2.11 (1.07–4.17, *p* = 0.032)	1.50 (0.71–3.17, *p* = 0.289)

## Discussion

LR after RN have been reported in up to 30% of patients with ccRCC [2]. Although other series reported recurrence rates lower than 2% after RN [7]. To date LRS, when feasible, represents the gold standard treatment. Nevertheless, the combination with systemic treatment has also been proposed [3]. Unfortunately, few historical studies, mostly based on small single-center experiences, with a low level of evidence are available on the efficacy of combined surgery and systemic therapy. We tested the hypothesis that adding systemic treatment to surgery might improve oncological outcomes in these patients. We relied on an international multicenter cohort including 96 patients. Our results have raised several interesting points of discussion.

First, our analyses showed a high rate of cancer-related deaths (up to 80.0%) in patients who had LR after RN. However, excellent cancer-specific survival at 3 years was achieved in those treated with LRS, with or without systemic therapy (92.3 and 63.2% respectively). Interestingly, after multivariable adjustment, the addition of systemic therapy to LRS did not lead to a statistically significant survival advantage compared to LRS alone. These results were independent of the effect of metastatic status at diagnosis, as confirmed by the interaction analysis. Our findings corroborate those of previous analyses showing no statistically significant beneficial effect of systemic therapies in addition to LRS [5, 7, 8, 12–14]. In particular, Bruno et al. reported results from a historical (1989–2004), small cohort of 34 patients with LR after RN. Of these 47.0% had evidence of metastases. Authors reported longer median survival in those who underwent LRS, irrespective of metastatic status. More specifically in M0 patients the median survival time was 71.4 vs. 9.9 months in those who had LRS vs. those who did not. Similarly, among M + patients the median survival was 16.3 vs. 11.8 months in those who had LRS vs. those who did not [12]. Moreover, Psutka et al. also relied on a historical (1970–2006) cohort of 63 patients who developed LR without (33/63) or with synchronous metastases (30/63) after RN [8]. Authors showed a survival advantage in patients receiving locally directed therapy when compared to those receiving systemic therapy alone or expectant management [8]. Interestingly, the authors also showed no statistically significant differences in terms of CSM after multivariable adjustment when comparing patients who underwent systemic therapy alone to those receiving expectant management [8]. Comparably, Margulis et al. also failed to show any statistically significant advantage of neoadjuvant or adjuvant systemic therapy at LRS in a cohort of 54 patients [7]. Surprisingly not even the use of targeted therapies had any advantage over immunotherapy with cytokines [7]. Furthermore, the most recent analysis by Du et al. also showed no statistically significant differences in terms of cancer-specific or overall survival when systemic targeted therapy was used [14]. Taken together our results, as well as those of previous works, suggest that LRS may offer the largest survival advantage either when used alone or in combination with systemic treatments. This survival advantage remains even in oligometastatic patients. However, our results also showed a clinically meaningful, even if not statistically significant, difference in terms of 3-year cancer-specific survival rates (almost 20%) when surgery is associated with systemic treatment. In consequence, we could speculate that patient selection may have a key role in a successful LR treatment.

Second, we explored the feasibility of LRS. Overall, in our study, only 16.0% of surgically treated patients had any complication and half of them had grade I complications according to the Clavien–Dindo classification. Our findings corroborate those of previous authors about surgery feasibility. In particular, Itano et al. reported data about 14 patients who underwent retroperitoneal exploration [5]. Of these 10 had complete en bloc excision of the RCC mass. The authors described postoperative complications in 33% of patients with no perioperative deaths. Of them, 2 patients were conservatively treated [5]. Low complication rates were also reported by Schrodter and colleagues; more specifically authors reported only one case of delayed wound healing due to a subcutaneous seroma [6]. Similar results were achieved by El Hajj et al. who reported complications in 22% of patients; mostly graded as Clavien–Dindo I [15]. In addition, Paparel et al. reported post-operative complications in 29.0% of surgically treated cases [13]. Importantly most of these complications were classified as Clavien–Dindo > 2 [13]. Furthermore, Du et al. reported intraoperative complications in 19.8% of patients and postoperative complications in 37.4% of patients. Most of the post-operative complications were classified as grade I or II (22.0%) [14]. Instead, higher complication rates were reported by Thomas and colleagues [10]. Authors showed postoperative complications in up to 45% of patients, although most of the complications were graded as Clavien–Dindo I or II [10]. Taken together these results suggest that LRS is feasible, but could be affected by serious complications, so careful patient selection is mandatory. To reduce the overall complication rates LRS should be performed only in high-volume centers after careful multidisciplinary evaluation. Unfortunately, literature has a lack of studies focusing on the effect of surgeon and center volume on LRS outcomes. However, we could expect better surgical outcomes in high-volume vs. low-volume centers, as have been previously reported for cytoreductive nephrectomy [16]. It is also worth considering the high variability among series in terms of complication reports. Such variation could be attributed to the retrospective nature of most of the available studies, reporting bias, as well as to the pathological characteristics of LR treated and the consequent selection bias. However, it should be highlighted that in our study no statistically significant differences were seen in terms of recurrence size or number of recurrences between surgically or not surgically treated patients.

Third, the presence of metastases at the time of diagnosis was associated with higher CSM rates. Nonetheless, in multivariable models adjusting for the treatment used, metastatic status was no longer an independent predictor of CSM. Such observation is of importance since those who were diagnosed as M + at the time of LR were more frequently treated with expected management (35.9% vs. 10.3%) or with systemic therapies only (43.6% vs.15.4%). The latter might represent undertreatment for these patients. Indeed, we may speculate that the cytoreductive effect of LRS might exert a survival advantage as have been reported for metastatic RCC treated with complete excision of the primary tumor and its metastatic sites [17–19]. Indeed, in patients that are free of any residual tumor systemic therapies could be delayed avoiding treatment-related adverse events [17, 18]. Similarly, a prospective phase 2 trial showed that patients with low metastatic burden can safely undergo surveillance [20], while another study showed that deferred treatment of metastatic patients after cytoreductive nephrectomy is also feasible [21]. This may be the case also for LR when a radical LRS has been performed [17, 18]. To the best of our knowledge evidence about the best timing for systemic treatment in patients with LR is lacking. Moreover, as pointed out by Dabestani et al. current systemic treatments have poor or no curative effect leading to further uncertainty about the best follow-up strategies to adopt in these patients [2]. Furthermore, the effect of immunotherapy should be also investigated in this setting [22]. Unfortunately, database granularity did not allow us to investigate the reasons why such large numbers of patients with M + only received expectancy management. In addition, the large timeframe, the absence of data about systemic therapies timing and the variety of treatment regimens used should also be acknowledged.

Our study is not devoid of limitations. First of all, its retrospective nature and the inherent risk of selection bias that could be residual even after multivariable adjustment for controlling confounders. Moreover, the relatively small cohort of patients included, with particular regard to those diagnosed as non-metastatic with isolated renal fossa recurrence, could also be considered as a major limitation of our study. Such limitation is shared with other relevant studies, as discussed above. Furthermore, even if the follow-up schedule was in accordance with the EAU guidelines, changes overtime in suggested protocols and the lack of imaging external control also represent a limitation. Future controlled studies with strict follow-up schedules and standardized systemic treatment regimens are still needed.

## Conclusion

Retroperitoneal recurrences after RN are associated with high CSM rates. Surgical treatment when feasible is recommended. Moreover, its association with a systemic treatment seems to warrantee adjunctive advantages in terms of survival, even in presence of metastases. But this association should be investigated in future controlled trials.

## Supplementary Information

Below is the link to the electronic supplementary material.

Supplementary file1 (DOCX 20 KB)

Supplementary file2 (DOCX 19 KB)

Supplementary file3 (DOCX 19 KB)
