# Supplementary material for: Management of local recurrence after radical nephrectomy: surgical removal with or without systemic treatment is still the gold standard. Results from a multicenter international cohort
Source: Int Urol Nephrol. 2021 Aug 21;53(11):2273–80. doi: 10.1007/s11255-021-02966-9 (PMC8494713; doi:10.1007/s11255-021-02966-9)
Supplement: Supplementary file 1 — Supplementary file1 (DOCX 20 KB) [file 11255_2021_2966_MOESM1_ESM.docx]

**Supplementary Table 1 - Baseline clinical characteristics at radical nephrectomy stratified according to metastatic status at recurrence**

|  | **M0**  **(n=52)** | **M+**  **(n=44)** | ***p-value*** |
| --- | --- | --- | --- |
| **Age** | 61.1 (50.7, 70.2) | 64.5 (56.0, 72.9) | *0.156^1^* |
| **Age according to tertile** |  |  | *0.087^2^* |
| - <55 yr | 20 (38.5%) | 8 (18.2%) |  |
| - 55-70 yr | 19 (36.5%) | 23 (52.3%) |  |
| - >70 yr | 13 (25.0%) | 13 (29.5%) |  |
| **Year of radical nephrectomy** |  |  | ***0.021^2^*** |
| - 1988-2004 | 22 (42.3%) | 29 (65.9%) |  |
| - 2005-2018 | 30 (57.7%) | 15 (34.1%) |  |
| **Male** | 34 (65.4%) | 29 (65.9%) | *0.957^2^* |
| **Charlson comorbidity index >1 (Missing=16)** | 9 (22.5%) | 5 (12.5%) | *0.239^2^* |
| **Laparoscopic radical nephrectomy approach** | 17 (32.7%) | 3 (6.8%) | ***0.002^2^*** |
| **Radical nephrectomy associated with adrenalectomy** | 20 (39.2%) | 21 (48.8%) | *0.349^2^* |
| **Lymph node dissection at RN** | 14 (27.5%) | 15 (34.1%) | *0.483^2^* |
| **Positive surgical margins at RN** | 5 (9.6%) | 2 (4.5%) | *0.341^2^* |
| **pT stage at radical nephrectomy** |  |  | *0.949^2^* |
| - T1-2 | 14 (26.9%) | 11 (25.0%) |  |
| - T3 | 33 (63.5%) | 28 (63.6%) |  |
| - T4 | 5 (9.6%) | 5 (11.4%) |  |
| **pN stage at radical nephrectomy** |  |  | *0.997^2^* |
| - pN0 | 12 (23.1%) | 10 (22.7%) |  |
| - pN1 | 8 (15.4%) | 7 (15.9%) |  |
| - pNx | 32 (61.5%) | 27 (61.4%) |  |
| **Non-ccRCC at RN specimen** | 16 (30.8%) | 11 (25.0%) | *0.531^2^* |
| **Tumor grade 3-4 (Missing =5)** | 40 (81.6%) | 30 (71.4%) | *0.249^2^* |
| **Sarcomatoid dedifferentiation at RN** | 4 (9.8%) | 3 (7.5%) | *0.718^2^* |
| **Necrosis at RN (Missing =4)** | 9 (18.8%) | 5 (11.4%) | *0.324^2^* |

1. Kruskal-Wallis rank sum test
2. Pearson’s Chi-squared test
