# Supplementary material for: Management of local recurrence after radical nephrectomy: surgical removal with or without systemic treatment is still the gold standard. Results from a multicenter international cohort
Source: Int Urol Nephrol. 2021 Aug 21;53(11):2273–80. doi: 10.1007/s11255-021-02966-9 (PMC8494713; doi:10.1007/s11255-021-02966-9)
Supplement: Supplementary file 2 — Supplementary file2 (DOCX 19 KB) [file 11255_2021_2966_MOESM2_ESM.docx]

**Supplementary Table 2 - Baseline clinical characteristics at recurrence stratified according to the metastatic status**

|  | **M0**  **(n=52)** | **M+**  **(n=44)** | ***p-value*** |
| --- | --- | --- | --- |
| **Recurrence size (cm) (Missing=21)** | 4.0 (2.8, 5.3) | 5.0 (3.0, 7.2) | *0.279^1^* |
| **Number of recurrences (Missing=2)** | 1.0 (1.0, 2.0) | 1.0 (1.0, 2.0) | *0.144^1^* |
| **Time to recurrence (months)** |  |  | *0.213^2^* |
| - <24 | 29 (55.8%) | 30 (68.2%) |  |
| - ≥24 | 23 (44.2%) | 14 (31.8%) |  |
| **Symptomatic at recurrence** | 4 (7.7%) | 3 (6.8%) | *0.870^2^* |
| **Recurrence in the renal fossa** | 20 (38.5%) | 28 (63.6%) | ***0.014^2^*** |
| **Recurrence at the psoas muscle** | 8 (15.4%) | 4 (9.1%) | *0.353^2^* |
| **Recurrence at ipsilateral adrenal gland** | 7 (13.5%) | 4 (9.1%) | *0.503^2^* |
| **Recurrence at lymph nodes** | 22 (42.3%) | 15 (34.1%) | *0.410^2^* |
| **Recurrence at other sites** | 2 (3.8%) | 5 (11.4%) | *0.158^2^* |
| **Systemic therapy after recurrence (Missing=18)** | 16 (41.0%) | 23 (59.0%) | *0.113^2^* |
| **Surgical approach to recurrence** |  |  | ***< 0.001^2^*** |
| - Open | 36 (69.2%) | 8 (18.2%) |  |
| - Laparoscopic | 5 (9.6%) | 1 (2.3%) |  |
| - No surgical treatment | 11 (21.2%) | 35 (79.5%) |  |
| **Type of treatment after recurrence (Missing=18)** |  |  | ***< 0.001^2^*** |
| - Combined | 10 (25.6%) | 6 (15.4%) |  |
| - Only medical | 6 (15.4%) | 17 (43.6%) |  |
| - Only surgery | 19 (48.7%) | 2 (5.1%) |  |
| - Expectant management | 4 (10.3%) | 14 (35.9%) |  |

1. Kruskal-Wallis rank sum test
2. Pearson’s Chi-squared test
