# Supplementary material for: Management of local recurrence after radical nephrectomy: surgical removal with or without systemic treatment is still the gold standard. Results from a multicenter international cohort
Source: Int Urol Nephrol. 2021 Aug 21;53(11):2273–80. doi: 10.1007/s11255-021-02966-9 (PMC8494713; doi:10.1007/s11255-021-02966-9)
Supplement: Supplementary file 3 — Supplementary file3 (DOCX 19 KB) [file 11255_2021_2966_MOESM3_ESM.docx]

**Supplementary Table 3 - Univariable and multivariable logistic regression models predicting metastatic status (M+ vs. M0) at recurrence**

|  | Univariable model  Odds Ratio (95%CI, p-value) | Multivariable model  Odds Ratio (95%CI, p-value) |
| --- | --- | --- |
| **Age at radical nephrectomy** |  |  |
| <55 years | Reference | Reference |
| 55-70 years | **3.03 (1.12-8.76, p=0.033)** | **3.02 (1.05-9.30, p=0.045)** |
| >70 years | 2.50 (0.83-7.97, p=0.110) | 3.40 (0.97-12.98, p=0.062) |
| **Year of radical nephrectomy (2005-2018 vs. 1988-2004)** | **0.38 (0.16-0.86, p=0.022)** | 0.55 (0.20-1.48, p=0.241) |
| **Time to recurrence** | 1.00 (0.99-1.01, p=0.783) | - |
| **Gender (Female vs. Male)** | 0.98 (0.42-2.28, p=0.957) | - |
| **Laparoscopic vs. open radical nephrectomy** | **0.15 (0.03-0.49, p=0.005)** | **0.21 (0.04-0.77, p=0.030)** |
| **pT stage at radical nephrectomy** |  |  |
| T1-2 | Reference |  |
| T3 | 1.08 (0.42-2.80, p=0.872) | - |
| T4 | 1.27 (0.29-5.70, p=0.748) | - |
| **pN stage at radical nephrectomy** |  |  |
| pN0 | Reference |  |
| pN1 | 1.05 (0.28-3.96, p=0.942) | - |
| pNx | 1.01 (0.38-2.75, p=0.980) | - |
| **Tumor grade at radical nephrectomy (3-4 vs. 1-2)** | 0.56 (0.20-1.50, p=0.252) | - |
| **Histotype at radical nephrectomy (non-ccRCC vs. ccRCC)** | 0.75 (0.30-1.84, p=0.532) | - |
| **Sarcomatoid dedifferentiation at RN (yes vs. no)** | 0.75 (0.14-3.63, p=0.719) | - |
| **Recurrence size** | 1.07 (0.93-1.25, p=0.355) | - |
| **Number of recurrences** | 1.57 (0.95-2.75, p=0.091) | - |
| **Recurrences at renal fossa (yes vs. no)** | **2.80 (1.24-6.54, p=0.015)** | 1.95 (0.77-4.97, p=0.160) |
